# Inspired to Garden: A Qualitative Study of Participants’ Experiences in an Academic Medical Center Garden

**DOI:** 10.7759/cureus.41695

**Published:** 2023-07-11

**Authors:** Heather Moore, Keelan Boisvert, Maria Bryan, Lisa Hoare, Michelle Gates, Bernice Garnett, Amanda G Kennedy, Michael Latreille

**Affiliations:** 1 Director, Shelburne Craft School, Shelburne, USA; 2 College of Arts and Sciences, University of Vermont, Burlington, USA; 3 Medicine, University of Vermont Medical Center, Burlington, USA; 4 Nutrition Services, University of Vermont Medical Center, Burlington, USA; 5 Executive Director, Vermont Garden Network, Essex Junction, USA; 6 College of Education and Social Services, University of Vermont, Burlington, USA; 7 Larner College of Medicine, University of Vermont, Burlington, USA

**Keywords:** patient experience, qualitative research, metabolic syndrome, horticulture therapy, gardening

## Abstract

Introduction

Gardening is a healthy activity that promotes nutrition and satisfaction, with positive impacts on patients with chronic diseases, including patients with obesity, diabetes, and cardiovascular disease. Hospital-based gardening programs may provide opportunities to introduce patients to gardening. However, few studies have included participant experience as a metric of evaluation. The objective of this study was to explore participant experience in a hospital-based gardening intervention designed for individuals with metabolic syndrome.

Methods

This study was a qualitative evaluation of free text responses from four questions included in post-participation questionnaires from 59 community-dwelling adults who participated in a hospital-based garden program located at the University of Vermont Medical Center in 2020 and 2021. Eligible participants included a convenience sample of novice gardeners with self-reported hypertension, diabetes, pre-diabetes, or overweight/obesity. We used an interpretative phenomenological approach to analyze the questionnaire data. The phenomenological cycle for each of the questions included: 1) reading and re-reading participant responses, 2) exploratory noting, 3) constructing experimental statements, 4) searching for connections across statements, and 5) naming the themes. This process also involved working with individual question-level themes to develop group themes across questions.

Results

This dataset was one of positivity about gardening, new information gleaned, and the quality of instruction. Several themes and codes emerged: program implementation (new knowledge, new skills, new connections, instructor ability, climate), self-efficacy (confidence, vicarious experience, mastery experience, verbal persuasion), and future change (behavior change, future issues/problem-solving, passing it on).

Conclusion

This study supports analyzing participant experience as part of hospital-based gardening interventions. We found positivity around program implementation, increased self-efficacy, and intentions to change behavior in ways that support healthy lifestyles.

## Introduction

Metabolic syndrome increases the risks of diabetes and cardiovascular disease and is defined as having at least three of the following factors, elevated waist circumference, elevated triglycerides, decreased high-density lipoprotein, elevated blood pressure, or elevated fasting glucose [1-4]. The prevalence of metabolic syndrome in the United States is estimated at nearly 35% [5,6]. A recent review estimated a quarter of the world population may be impacted by metabolic syndrome [7].

Gardening is often sought for the enjoyment and promotion of mental and physical health [8,9]. Gardening has been found to have a wide range of health benefits, including the reduction of sedentary lifestyles in patients with diabetes, and a beneficial association with cardiovascular disease risk factors [10,11]. In an analysis of 22 case studies investigating horticultural therapy or structured gardening [12], there was a significant positive association between gardening and body mass index (BMI), mood, life satisfaction, quality of life, and sense of community [13]. A systematic review of 12 studies focused on urban gardening found a variety of benefits, including better access to healthy food, greater value placed on eating and cooking healthy food, and improved eating habits [14]. In an observational study examining the impact of a community gardening program on BMI, gardeners in the program were found to have significantly lower BMI than their non-gardening neighbors, siblings, and spouses [15].

Gardens specifically located on hospital grounds are increasingly commonplace and are employed for a number of reasons, including physical therapy [16], support for pregnant women and their partners [17], and to treat individuals with metabolic or cardiovascular disease [18]. Feasibility, including acceptability, demand, and willingness to engage in gardening activities in a hospital-based program is supported by a small study examining individuals with cardiovascular risk factors [18].

Acknowledging the proven and theoretical benefits of gardening for conditions associated with metabolic syndrome along with evidence supporting feasibility, a gap remains in understanding whether, or how, a brief intervention might create lasting behavior change. Giving participants the opportunity to describe the health impacts of a gardening program in their own words would provide a better understanding of how they perceive, and potentially change from, their time in a hospital-based gardening program, tease out the most impactful elements of a program, and inform future interventions. In this phenomenological study, we sought to employ qualitative research methodologies to investigate the impacts of a brief, structured gardening intervention for participants with metabolic syndrome.

## Materials and methods

The Gardening for Health (G4H) program took place at the University of Vermont Medical Center (UVMMC), a 620-bed academic medical center located in Burlington, Vermont, that serves over one million patients in Vermont and northern New York. The program was a partnership between the non-profit, Vermont Garden Network (VGN), and UVMMC, supported with funding from the UVM Medical Center Foundation. The program was offered free of charge to eligible participants, which included a convenience sample of community-dwelling novice gardeners with self-reported hypertension, diabetes, pre-diabetes, or overweight/obesity. Participants were recruited from UVMMC primary care clinics, community flyers, a press release, and on the UVMMC website. The garden space is adjacent to the main hospital, includes an Americans with Disabilities Act (ADA)-accessible ramp, and encompasses 4,000 square feet, including 450 square feet of participant-raised beds flanked by 750 square feet of additional gardening space for demonstrations and harvesting.

The foundation of the Gardening for Health (G4H) program was a combination of sessions in the garden alternating with at-home workshops. Hands-on garden sessions were held every other week, initially running for eight weeks when G4H was introduced in 2020, and extended to nine weeks, with the addition of a fifth garden session in 2021 in order to end in the garden rather than with an at-home workshop. Each on-site session followed a similar schedule, beginning with a stretching routine, followed by meditation, didactic gardening instruction, hands-on gardening, nutrition instruction paired with recipe tasting, and finally harvesting.

Each participant was assigned a raised bed in which to learn and experiment with a range of seeds and plants. Two co-authors (LH and MG) demonstrated gardening skills, after which participants were invited to participate. For instance, after receiving instructions on seed spacing and depth, participants would plant seeds in their raised beds. Participants were encouraged to ask questions and educators emphasized resiliency in the face of the unexpected. Each session highlighted a different topic necessary for successful gardening from start to finish, including garden planning, sowing seeds, planting seedlings, garden maintenance, pest control, harvesting produce, and food preservation. Culinary, nutritional, and mindfulness elements of in-person sessions aimed to reinforce the connections between these concepts and health.

Structured at-home gardening workshops were designed to require no specialized equipment or space, and participants were given all materials needed to complete them. The intent of these workshops was to engage participants in the time between garden sessions and foster independence with gardening concepts. Examples included potting an herb or starting a tray of pea shoots from seed. Participants were encouraged to partake in other health-related activities or exercises on their own. These included health education videos available online, self-guided nature walks, stretching exercises, or trying a new recipe. A companion notebook included all gardening and nutritional curricula presented in the garden sessions, as well as recipes and all of the recommended activities for at-home workshops, with space for notes.

Program data for qualitative evaluation was collected through post-participation questionnaires using Research Electronic Data Capture (REDCap) electronic data capture tools hosted at UVM [19] within a week of program completion. The four free text questions available for analysis included: How has your understanding or perception of gardening changed since the beginning of the program? Would you recommend this program to a friend? Did this program meet your expectation (why/how)? What challenges, if any, do you expect in your ability to garden in the future? This project was approved as exempt research by the University of Vermont Committee on Human Research (STUDY00001046). Completing the post-participation questionnaire was considered implied consent.

To investigate participant perceptions of G4H, we posed the following research questions: How did participants describe the essence of their experience in G4H? What (if any) themes emerge to demonstrate participants' growth of confidence/self-efficacy in gardening as a result of the G4H intervention? What (if any) themes emerge to demonstrate the possibility of participants making healthy changes or planning to make healthy changes in their lives?

The approach to analysis relied heavily on an interpretative phenomenological approach (IPA) [20] but was modified to suit the scope of the data set. IPA is “phenomenological in that it is concerned with exploring experience in its own terms” [20]. We chose this qualitative approach because we were most interested in the essence of the experience-the changes or effects individuals in the intervention described as resulting from their G4H workshop series. Although IPA typically employs interview data, this technique has proven effective in utilizing other types of data, including surveys, in order to gain insight into participant experience [21,22].

We analyzed the qualitative data from two participant sets, 2020 and 2021, by question and not by year, in order to keep the answers within the context of the questions asked on the G4H survey. The phenomenological cycle for each of the questions included: 1) reading and re-reading participant responses, 2) exploratory noting, 3) constructing experimental statements, 4) searching for connections across statements, and 5) naming the themes. This process also involved working with individual question-level themes to develop group themes across questions.

This approach was followed by a priori codes drawn from Bandura’s self-efficacy construct [23] to assess evidence of increased efficacy and evidence of behavior change. We explored the lived experience of those participating in the G4H intervention to better understand how best this intervention can support individuals with metabolic syndrome.

In addition to being reflective in the process and observing our own biases, we also engaged three other trustworthiness strategies, including double-coding, member checking, and triangulation [23]. The analysis was primarily conducted by HM and double-coded by KB. Initial double coding resulted in 96% consistency. After an open discussion, this intercoder reliability increased to 100%.

HM was a Doctor of Education in Educational Leadership and Policy Studies student at the time of this study and worked under the direction of BG, an experienced investigator. The G4H participants did not have a relationship with the co-authors who conducted the analyses. Data were analyzed by hand without software. 

Because the sample size is relatively large for a qualitative study, with short impressions from many people rather than lengthy interview-based responses from relatively few, and because we combined an IPA method with a second cycle of a priori coding, we utilized data saturation. We used data saturation to mean that we reached a point in the analysis where no new information was found and repetition of themes/codes signaled a strong pattern across experience [24].

## Results

A total of 45 individuals completed the program in 2020 and 38 in 2021. The age range in the combined 2020 and 2021 groups was 31 to 79 years. Participants identified predominantly as female (82.1%), white (80.6%), and college-educated (57.6%). Slightly less than half of the participants (43%) were employed full time and 25.4% were retired. Eighty percent of participants reported being overweight, 50.8% reported having high blood pressure, and 43.3% reported having diabetes or prediabetes. A total of 59 individuals completed the qualitative portion of the post-participation questionnaire, including 34 individuals from 2020 (76%) and 25 individuals from 2021 (66%).

Overall, this dataset reflected positivity and excitement about gardening, the new information gleaned through the program, and the quality of instruction. To present disconfirming evidence, we noted that a few individuals commented negatively on the intervention. “Don't think you can do anything about the folks that overshare their [sic] comments, but it can be annoying.” Concerns about parking were referenced more than any other issue. Negative comments were rare in the data set (4%).

The positivity of the data specifically relates to the themes that emerged through our analysis: program implementation, self-efficacy, and future change (Table 1). All three themes contribute to answering the first research question, “How did participants describe the essence of their experience in Gardening for Health?” Self-efficacy answers the research question, “What (if any) themes emerge to demonstrate participants' growth of confidence/self-efficacy in gardening as a result of the Gardening for Health intervention?” Finally, future change answers the research question, “What (if any) themes emerge to demonstrate the possibility of participants making healthy changes or planning to make healthy changes in their lives?”

**Table 1:** Visual representation of findings from an analysis of 59 questionnaires from participants in a hospital-based gardening program

Theme/Codes	Description / Example Quotes
Theme One. Program Implementation	Refers to effective creation and production of programming and resulting knowledge gained by participants
New Knowledge: new learning or understanding	“Gained a new understanding of how to relate to gardening and eating from your garden in a fun and interesting (and informed) way”
New Skills: skills or techniques learned from the intervention	“I learned how to successfully grow from seeds and transplanted vegetables.”
New Connections: connecting healthy food/nutrition and gardening	“Before I saw gardening just as a hobby, now I see it as a way to get healthier and safer products for my body.”
Instructor Ability: positive affirmation of instructors in the intervention	“The wonderful group who managed it, showed how rewarding, interesting, and fun gardening can be, in addition to sharing their immense knowledge and experience with us in a way that was very accessible.”
Climate: discussion of a comfortable learning environment	“The atmosphere was warm and welcoming, and no one felt out of place or afraid to ask questions.”
Theme Two: Self-efficacy	Refers to new confidence in healthy eating or gardening gained from the intervention and explores this confidence within the context of Bandura’s self-efficacy construct [23]
Confidence: increased confidence in gardening or other aspect of the intervention	“I am so much more confident with my garden, which makes me far more motivated to be active in it.”
Vicarious Experience: evidence of modeling by another individual	“Having the lettuce already started gave me the sense that growing things didn't take forever ;D”
Mastery Experience: provides feedback from one’s successes and also failures of an experience/performing tasks	“I can now grow vegetables in a planter box . Grew basil from transplant. Grew beets and spinach from seeds”
Verbal Persuasion: verbal feedback from others	“Due to the encouragement of presenters and their inspiring efforts to relay the possibility for healthy eating improvements, I’m more motivated to pay attention to what I eat.”
Theme Three: Future Change	Refers to how this intervention may have effects on participants as well as their community in the future
Behavior Change: participant indicated they had changed or would change a behavior which should result in better health	“I also found myself motivated to eat more fresh food and incorporate different vegetables into breakfast and snacks where I might not have opted to prior to taking this class.”
Future Issues/Problem Solving: participant described possible future issues with regard to gardening in the future (may have included problem solving/solution to that issue)	“Physical challenges- but I’m working on those and hope to be in better physical condition by next summer. There may also be financial challenges- as a senior living on social security alone, the cost of gardening may exceed my ability to pay for what I want… but I believe in starting small and adding a variety of plants whenever possible”
Passing it on: indication of sharing knowledge or outcomes of course with others	“I hope to pass on to my grandchildren.”

Theme one: program implementation

The most saturated set of codes in this data set involved those which aligned with program implementation. Program implementation refers to the effective creation and production of programming and the resulting knowledge gained by participants. The codes of this theme included new knowledge, new skills, new connections, instructor ability, and climate.

Within this theme, the code new knowledge heavily saturated the data set, with individuals indicating that they had learned from the intervention; often, these responses emphasized great amounts of learning, content, or information gleaned. “Gardens are adaptable with care and a lot of food diversity can be taken with little effort in our diets.” The code new skills indicate that an individual learned techniques that enabled them to be a gardener or better gardener or to cook and eat healthier foods. This code demonstrates that something specific has increased the participant’s skillset in healthy eating/gardening. “It was wonderful learning how to prepare foods in a different way, like roasting radishes instead of eating them raw which I hate. I love them roasted! Also, different food hacks like substituting applesauce for oils, and making mock chicken salad with garbanzo beans. Eating Bee Balm petals!” The code new connections indicate that the participant sees a new relationship between ideas, usually between nutrition and gardening, or gardening and exercise. This code indicates that the intervention allowed the participant to view their health landscape differently. “Yes, before I saw gardening just as a hobby, now I see it as a way to get healthier and safer products for my body.” Instructor ability refers to positive proclamations about the G4H instructors. Participants shared how the instructors were able to impart knowledge effectively or their enjoyment of the learning facilitated by the instructors. “The facilitators were wonderful people, full of knowledge and desire to impart information.” Lastly, the code climate refers to the learning environment being comfortable or conducive to questions asked by participants. “The atmosphere was warm and welcoming, and no one felt out of place or afraid to ask questions.”

Theme two: self-efficacy

Self-efficacy refers to a new confidence in healthy eating or gardening gained from the intervention and explores this confidence within the context of Bandura’s self-efficacy construct [23]. The codes of this theme included confidence, vicarious experience, mastery experience, and verbal persuasion.

The code for confidence indicates that a participant’s confidence increased as a result of the intervention. Often, participants indicated that they were, or would be, gardening and/or cooking healthier foods. “I was going to give up and not have a garden next year, but now I have more tools to use and have decided to keep on carrying on.” The subcodes of Bandura’s self-efficacy construct indicate vicarious experience, mastery experience, and verbal persuasion. Vicarious experience indicates that participants watched the instructors perform tasks in the garden. “Having the lettuce already started gave me the sense that growing things didn't take forever.” Mastery experience or performance feedback indicates that the participant was able to grow their own vegetables and cook their own food. “I can now grow vegetables in a planter box. Grew basil from transplant. Grew beets and spinach from seeds.” Verbal persuasion indicates that the participant received verbal feedback from the instructors as part of the intervention. “Due to the encouragement of presenters and their inspiring efforts to relay the possibility for healthy eating improvements, I’m more motivated to pay attention to what I eat.” Physiological feedback, another part of Bandura’s self-efficacy construct, did not appear in this data set.

Theme three: future change 

The future change theme details how this intervention may have effects on participants as well as their community in the future. The codes of this theme included behavior changes, potential issues in the future, and solving those issues, as well as how participants may pass on the information gleaned in this intervention.

Overall, the final set of codes related to how individuals wrote about the impact of the intervention. This theme became wider than the original question as it details how the intervention may cause a change in other individuals’ lives as well. That is, it included healthy changes that individuals may be creating in their social circles and families, for instance cooking more vegetables for their children. There were two behavior change codes indicating that the participant had changed or would change behavior which should result in better health. The first, healthy behavior change: consumption, indicates a healthy change in eating. “This program restarted my love of homegrown food with the simplest of recipes. My kitchen has turned into a chemistry lab of herbs, spices, fruits and vegetables, seeds, and nuts being used in [place of] butter, salts, sugars, soups, salads, eggs, meats, and smoothies. Just the ideas used in the classes have sparked more ideas in my kitchen.” The second, healthy behavior change: gardening, indicates a healthy change with regard to gardening. “Growing my own food is so rewarding and much healthier.” Future issues/problem-solving indicates that the participant stated problems or issues that may impede gardening in the future and then proposed a way to mitigate or solve that problem. “I think weather and watering are always a factor/challenge, but I got good suggestions and look forward to watering more efficiently.” The passing it-on code indicates that the participants would recommend the intervention or aspects of the intervention to other individuals. “I have recommended it to two people already and told them how to access the website.” Individuals also discussed how they would share the knowledge that they gained in the intervention with others. “I hope to pass it on to my grandchildren.”

## Discussion

This study was exploratory and, as such, sought qualitative data in an open-ended fashion rather than in specific areas such as self-efficacy or behavior change. Nonetheless, our findings were rich with codes and themes pertaining to implementation fidelity, positivity, instructor ability, creation of new knowledge, and self-efficacy, as well as future projections of healthy behavior change and passing on aspects of learning to others in the household or community. Participants also simply enjoyed the program, which is worth noting, as interventions conventionally considered to address health habits are not uniformly greeted with such enthusiasm.

Much of the data suggested that participants found the teaching methods effective. Numerous factors likely contributed to this high degree of fidelity. Gardening is a longitudinal process requiring a diverse set of skills employed at different times and thus lends itself to multiple types of teaching. The team utilized many teaching methods, including demonstration of techniques, didactic teaching, hands-on learning, recipe tastings, and guided mindfulness. At-home workshops and a companion notebook, containing much of the content of the intervention, likely furthered engagement and retention by reinforcing concepts and techniques, and by making content accessible for review. This overall diversity of content and delivery mirrors other studies that use gardening as an intervention for health behavior change [18].

The finding of increased self-efficacy has important implications, as this is considered a key component to behavior change and also to self-management across a wide spectrum of conditions including sedentariness [25], hypertension [26], and diabetes [27]. It has been similarly studied as a potential indicator of healthy behavior changes in other gardening interventions [28]. In our program, instructors emphasized throughout the workshops how to work toward better predictability and controllability in the garden, two factors that are considered conducive to the enhancement of efficacy beliefs [23].

Conceptually, future behavior change is supported by the incorporation of knowledge and improved self-efficacy outlined in the first two themes, but the direct evidence that emerged in this study is important, as a durable impact on behavior is the ultimate goal of any lifestyle intervention. Perhaps equally promising is the suggestion that knowledge and positive habits are passed through families and communities. This has been noted in other gardening studies and implies the possibility of an important multiplicative effect for a program that is naturally constrained in scalability [15,29].

This study has several important limitations. These data, and the wider published literature, are subject to selection bias, as individuals either willing to participate in such a program or who are already engaged in gardening do so by choice and usually out of self-motivated interest. The overwhelmingly positive results published in the literature suggest that there may also be publication bias. Methodologically, several factors may have limited the depth and richness of the data we obtained. While questionnaires have been used with some success in similar research [21,22], qualitative phenomenological studies typically deal with deeper data sets generated by interviews. Responses were further constrained by the small number of open-response questions included in our instrument. While robust, our findings must be contextualized in the inherent limitations of the tool utilized.

Although we were interested in the experience of participants, we did not specifically focus on behavior change or self-efficacy in the creation of our questionnaire. Questions aimed at qualitative data were limited, brief, and completely open-ended. The benefit of this approach is that unprompted responses might be considered less biased, and also that room is left for participants to respond in unanticipated domains, providing avenues for future study. The downside is a lack of depth in areas either proven or thought to be of high yield. Future studies should include more in-depth questions in areas of interest and ideally pair those with interviews to better understand the experience of participants.

Finally, it is worth noting that the expressed intent to change behavior remains several steps removed from health outcomes such as reduced body mass index, improved blood pressure, or glycemic control and reduction of adverse health outcomes such as myocardial infarction or stroke. Confirmation of behavior change through longitudinal assessment of participants would be informative.

## Conclusions

This study demonstrates that the G4H program was met with positivity and enthusiasm by participants. The intervention resulted in new knowledge, increased confidence in gardening and healthy eating, as well as an indication of planned future steps toward gardening and new healthy behaviors. While we focused on participants with metabolic syndrome, our findings can likely be applied more broadly to other populations. We expect that this study may serve as a model to better understand how participants experience a gardening intervention and what they plan to carry into their future.
